# Effectiveness of home visiting programs on child outcomes: a systematic review

**DOI:** 10.1186/1471-2458-13-17

**Published:** 2013-01-09

**Authors:** Shelley Peacock, Stephanie Konrad, Erin Watson, Darren Nickel, Nazeem Muhajarine

**Affiliations:** 1 College of Nursing, University of Saskatchewan, 414 St. Andrew’s College, 1121 College Drive, Saskatoon, SK S7N 0W3, Canada; 2 Department of Community Health & Epidemiology, University of Saskatchewan, Saskatoon, SK, Canada; 3 Health Sciences Library, University of Saskatchewan, Saskatoon, SK, Canada; 4 Physical Medicine & Rehabilitation, College of Medicine, University of Saskatchewan, Saskatoon, SK, Canada; 5 Saskatchewan Population Health and Evaluation Research Unit, Saskatoon, SK, Canada

**Keywords:** Systematic review, Paraprofessionals, Young children, Home visits, Socially high-risk families

## Abstract

**Background:**

The effectiveness of paraprofessional home-visitations on improving the circumstances of disadvantaged families is unclear. The purpose of this paper is to systematically review the effectiveness of paraprofessional home-visiting programs on developmental and health outcomes of young children from disadvantaged families.

**Methods:**

A comprehensive search of electronic databases (e.g., CINAHL PLUS, Cochrane, EMBASE, MEDLINE) from 1990 through May 2012 was supplemented by reference lists to search for relevant studies. Through the use of reliable tools, studies were assessed in duplicate. English language studies of paraprofessional home-visiting programs assessing specific outcomes for children (0-6 years) from disadvantaged families were eligible for inclusion in the review. Data extraction included the characteristics of the participants, intervention, outcomes and quality of the studies.

**Results:**

Studies that scored 13 or greater out of a total of 15 on the validity tool (*n* = 21) are the focus of this review. All studies are randomized controlled trials and most were conducted in the United States. Significant improvements to the development and health of young children as a result of a home-visiting program are noted for particular groups. These include: (a) prevention of child abuse in some cases, particularly when the intervention is initiated prenatally; (b) developmental benefits in relation to cognition and problem behaviours, and less consistently with language skills; and (c) reduced incidence of low birth weights and health problems in older children, and increased incidence of appropriate weight gain in early childhood. However, overall home-visiting programs are limited in improving the lives of socially high-risk children who live in disadvantaged families.

**Conclusions:**

Home visitation by paraprofessionals is an intervention that holds promise for socially high-risk families with young children. Initiating the intervention prenatally and increasing the number of visits improves development and health outcomes for particular groups of children. Future studies should consider what dose of the intervention is most beneficial and address retention issues.

## Background

Caring for infants and young children can be challenging for many parents; it can be further complicated when families are poor, lack social support, or have addiction problems [1]. Home visiting (HV) programs attempt to address the needs of these at-risk families with young children by offering services and support that they might not otherwise access. Home visiting programs have been in existence now for more than 20 years [2]. The benefit of HV programs is that the service is brought to socially isolated or disadvantaged families in their own homes and as such, may increase their sense of control and comfort, allowing them to get the most benefit from services offered. Also offering the programs in the home environment allows home visitors to provide a more tailored approach to service delivery [2,3].

HV programs, however, have difficulties to overcome in order to deliver services. Target families may not accept enrolment into a program or when they do agree may later elect not to begin the program [3]. Some possible explanations for this include the facts that home visitors may be viewed as intruding or because families may find it difficult to open their homes to home visitors. Achieving consistency in program delivery can also be difficult; families may not receive the planned number of visits, and visitors may not deliver the content according to the program model [3]. Despite these challenges, the benefits of HV programs outweigh the limitations. To achieve the aims of HV programs it is important that they be shaped by the community and families they serve and that their outcomes be evaluated routinely as part of program improvement.

There are a number of systematic reviews and meta-analyses that explore the effectiveness of HV programs with disadvantaged families [4-7], many of which focus on the prevention of child maltreatment [8-10]. We were not able to locate a systematic review that focused on the delivery of HV programs by paraprofessionals and the effect this method of service delivery has on children’s developmental and health outcomes, so we decided to conduct one to fill this gap in the literature. This work is important to policy-makers and program planners in that these types of programs may be desirable in regions where the higher costs associated with nurse-led HV interventions mean that they are not a feasible option.

## Methods

The research question for this systematic review was: *What is the effectiveness of paraprofessional HV programs in producing positive developmental and health outcomes in children from birth to six years of age living in socially high-risk families?* For the purposes of this review, a paraprofessional is an individual delivering an HV program whose credentials do not include clinical training (e.g., developmental psychologist, etc.) and who is not licensed. Socially high-risk families are those who live in poor economic circumstances, receive government assistance or who have inadequate income to meet the needs of the family. We chose broad outcome measures to make the review wide-ranging. These definitions are reflective of the types of programs, families, and research being done with HV programs across North America and elsewhere.

### Literature search strategies

An experienced health sciences librarian searched the CINAHL PLUS, Cochrane Library, ProQuest Dissertations and Theses, EMBASE, MEDLINE, PSYCINFO and Sociological Abstracts databases. Weekly alerts from all databases except Cochrane Library were set up to allow inclusion of newly published articles. Where possible, results were limited to the English language with a publication date of 1990 or later (up to May 2012).

### Assessment of studies using relevance and validity tools

The tools utilized for assessing the relevance and quality of the studies were based on previously developed tools [11,12]. Article titles and abstracts, when available, were screened by one reviewer to determine whether they might meet eligibility criteria: 1) publication date on or after 1990; 2) written in English; 3) involving an evaluation of an HV program delivered by paraprofessionals; 4) study population of mothers and/or children (0-6) from socially high-risk families; 5) including one of the following outcomes: birth, perinatal, developmental, health and/or risk for occurrences of child abuse/neglect; and 6) incorporation of a control group, pretest/post-test design or quasi-experimental design. A principal reviewer assessed all the papers, and one of two secondary reviewers independently evaluated their relevance, with a third to adjudicate if needed. When necessary, we contacted researchers to clarify components of their research.

Relevant articles were then evaluated to determine the research quality using a validity tool with five items with scores ranging from 0-3, for a total maximum quality score of 15. The tool assessed studies based on how well they addressed potential biases, through assessment of the: (a) *design/allocation to intervention* (e.g., random assignment {3}, matched cohort {1}); (b) *attrition of complete sample* (e.g., <17% {3}, >33% {0}); (c) *control of confounders* (e.g., controlled through RCT design {3}, no evidence of controlling {1}); (d) *measurement tools* (e.g., well-described/pre-tested tools and blinded data assessors {3}, lack of pretesting and blinding {1}); and (e) *type and appropriateness of statistical analysis* (e.g., multivariate analysis {3}, descriptive analysis {1}). Two reviewers independently assessed the quality and discussed articles to reach consensus when discrepancies occurred.

### Data extraction

We performed data extraction on high-quality studies (i.e., those scoring 13 or greater out of a possible 15), using these categories: (a) study design; (b) purpose or problem; (c) sample details; (d) intervention frequency, duration and provider; (e) instrument(s)/measures utilized; and (f) results and implications of the study. This process was done independently by three reviewers, consulting with each other when necessary.

### Data synthesis

We used descriptive synthesis to summarize the characteristics of the participants, intervention, outcomes, and quality of the included studies, based on data extracted. Due to the diversity of the outcomes included in the studies, varying types of statistical analysis conducted, and measures of associations reported, calculation of overall summary estimates (i.e., meta-analysis) was not possible. An alpha level of 0.05 was considered statistically significant for the purposes of this review.

## Results and discussion

### Literature search

By using broad search criteria (in order to locate as many potential articles as possible) we identified 2939 records through database searches, which were reduced to 2088 records after duplicates were removed. We found an additional 18 articles by searching the reference lists of all potentially relevant studies. Email alerts resulted in a review of an additional 145 articles, resulting in a total of 2233 articles reviewed. Please see Table 1 that contains a sample of the initial search strategy employed.

**Table 1:** Initial OVID MEDLINE search strategy (1950-present, searched March 2011)

1	domicil*.mp. [mp = title, original title, abstract, name of substance word, subject heading word, unique identifier]
2	residential.mp. [mp = title, original title, abstract, name of substance word, subject heading word, unique identifier]
3	home?.mp. [mp = title, original title, abstract, name of substance word, subject heading word, unique identifier]
4	dwelling?.mp. [mp = title, original title, abstract, name of substance word, subject heading word, unique identifier]
5	lodging?.mp. [mp = title, original title, abstract, name of substance word, subject heading word, unique identifier]
6	housing/
7	housing.mp. [mp = title, original title, abstract, name of substance word, subject heading word, unique identifier]
8	house?.mp. [mp = title, original title, abstract, name of substance word, subject heading word, unique identifier]
9	residence?.mp. [mp = title, original title, abstract, name of substance word, subject heading word, unique identifier]
10	1 or 2 or 3 or 4 or 5 or 6 or 7 or 8 or 9
11	visit*.mp. [mp = title, original title, abstract, name of substance word, subject heading word, unique identifier]
12	house calls/
13	house call?.mp. [mp = title, original title, abstract, name of substance word, subject heading word, unique identifier]
14	intervention?.mp. [mp = title, original title, abstract, name of substance word, subject heading word, unique identifier]
15	home care services/
16	11 or 12 or 13 or 14 or 15
17	10 and 16
18	exp infant/
19	infan*.mp. [mp = title, original title, abstract, name of substance word, subject heading word, unique identifier]
20	baby.mp. [mp = title, original title, abstract, name of substance word, subject heading word, unique identifier]
21	babies.mp. [mp = title, original title, abstract, name of substance word, subject heading word, unique identifier]
22	toddler?.mp. [mp = title, original title, abstract, name of substance word, subject heading word, unique identifier]
23	preschooler?.mp. [mp = title, original title, abstract, name of substance word, subject heading word, unique identifier]
24	pre-schooler?.mp. [mp = title, original title, abstract, name of substance word, subject heading word, unique identifier]
25	exp child/
26	child*.mp. [mp = title, original title, abstract, name of substance word, subject heading word, unique identifier]
27	newborn?.mp. [mp = title, original title, abstract, name of substance word, subject heading word, unique identifier]
28	neonate?.mp. [mp = title, original title, abstract, name of substance word, subject heading word, unique identifier]
29	neo-nate?.mp. [mp = title, original title, abstract, name of substance word, subject heading word, unique identifier]
30	neonatal.mp. [mp = title, original title, abstract, name of substance word, subject heading word, unique identifier]
31	neo-natal.mp. [mp = title, original title, abstract, name of substance word, subject heading word, unique identifier]
32	prenatal.mp. [mp = title, original title, abstract, name of substance word, subject heading word, unique identifier]
33	pre-natal.mp. [mp = title, original title, abstract, name of substance word, subject heading word, unique identifier]
34	prenatal care/
35	postnatal care/
36	perinatal care/
37	antenatal.mp. [mp = title, original title, abstract, name of substance word, subject heading word, unique identifier]
38	ante-natal.mp. [mp = title, original title, abstract, name of substance word, subject heading word, unique identifier]
39	postnatal.mp. [mp = title, original title, abstract, name of substance word, subject heading word, unique identifier]
40	post-natal.mp. [mp = title, original title, abstract, name of substance word, subject heading word, unique identifier]
41	postpartum.mp. [mp = title, original title, abstract, name of substance word, subject heading word, unique identifier]
42	post-partum.mp. [mp = title, original title, abstract, name of substance word, subject heading word, unique identifier]
43	exp pregnancy/
44	pregnan*.mp. [mp = title, original title, abstract, name of substance word, subject heading word, unique identifier]
45	expectant.mp. [mp = title, original title, abstract, name of substance word, subject heading word, unique identifier]
46	expecting.mp. [mp = title, original title, abstract, name of substance word, subject heading word, unique identifier]
47	perinatal.mp. [mp = title, original title, abstract, name of substance word, subject heading word, unique identifier]
48	peri-natal.mp. [mp = title, original title, abstract, name of substance word, subject heading word, unique identifier]
49	exp parents/
50	parent*.mp. [mp = title, original title, abstract, name of substance word, subject heading word, unique identifier]
51	mother*.mp. [mp = title, original title, abstract, name of substance word, subject heading word, unique identifier]
52	father*.mp. [mp = title, original title, abstract, name of substance word, subject heading word, unique identifier]
53	matern*.mp. [mp = title, original title, abstract, name of substance word, subject heading word, unique identifier]
54	patern*.mp. [mp = title, original title, abstract, name of substance word, subject heading word, unique identifier]
55	18 or 19 or 20 or 21 or 22 or 23 or 24 or 25 or 26 or 27 or 28 or 29 or 30 or 31 or 32 or 33 or 34 or 35 or 36 or 37 or 38 or 39 or 40 or 41 or 42 or 43 or 44 or 45 or 46 or 47 or 48 or 49 or 50 or 51 or 52 or 53 or 54
56	17 and 55
57	poverty/
58	poverty.mp. [mp = title, original title, abstract, name of substance word, subject heading word, unique identifier]
59	poor.mp. [mp = title, original title, abstract, name of substance word, subject heading word, unique identifier]
60	disadvantaged.mp. [mp = title, original title, abstract, name of substance word, subject heading word, unique identifier]
61	vulnerable populations/
62	vulnerable.mp. [mp = title, original title, abstract, name of substance word, subject heading word, unique identifier]
63	low-income.mp. [mp = title, original title, abstract, name of substance word, subject heading word, unique identifier]
64	at-risk.mp. [mp = title, original title, abstract, name of substance word, subject heading word, unique identifier]
65	high-risk.mp. [mp = title, original title, abstract, name of substance word, subject heading word, unique identifier]
66	low ses.mp. [mp = title, original title, abstract, name of substance word, subject heading word, unique identifier]
67	low socioeconomic.mp. [mp = title, original title, abstract, name of substance word, subject heading word, unique identifier]
68	low socio-economic.mp. [mp = title, original title, abstract, name of substance word, subject heading word, unique identifier]
69	lower class.mp. [mp = title, original title, abstract, name of substance word, subject heading word, unique identifier]
70	social welfare/
71	welfare.mp. [mp = title, original title, abstract, name of substance word, subject heading word, unique identifier]
72	social assistance.mp. [mp = title, original title, abstract, name of substance word, subject heading word, unique identifier]
73	government assistance.mp. [mp = title, original title, abstract, name of substance word, subject heading word, unique identifier]
74	low* income.mp.
75	indigent?.mp.
76	indigenc?.mp. [mp = title, original title, abstract, name of substance word, subject heading word, unique identifier]
77	57 or 58 or 59 or 60 or 61 or 62 or 63 or 64 or 65 or 66 or 67 or 68 or 69 or 70 or 71 or 72 or 73 or 74 or 75 or 76
78	56 and 77
79	paraprofessional?.mp. [mp = title, original title, abstract, name of substance word, subject heading word, unique identifier]
80	para-professional?.mp. [mp = title, original title, abstract, name of substance word, subject heading word, unique identifier]
81	lay.mp. [mp = title, original title, abstract, name of substance word, subject heading word, unique identifier]
82	peer?.mp. [mp = title, original title, abstract, name of substance word, subject heading word, unique identifier]
83	nonprofessional?.mp. [mp = title, original title, abstract, name of substance word, subject heading word, unique identifier]
84	non-professional?.mp. [mp = title, original title, abstract, name of substance word, subject heading word, unique identifier]
85	mentors/
86	mentor*.mp. [mp = title, original title, abstract, name of substance word, subject heading word, unique identifier]
87	visitor*.mp. [mp = title, original title, abstract, name of substance word, subject heading word, unique identifier]
88	79 or 80 or 81 or 82 or 83 or 84 or 85 or 86 or 87
89	78 and 88
90	limit 89 to (english language and yr = "1990 - 2010")

### Relevance and validity tool assessment

**Figure 1:**
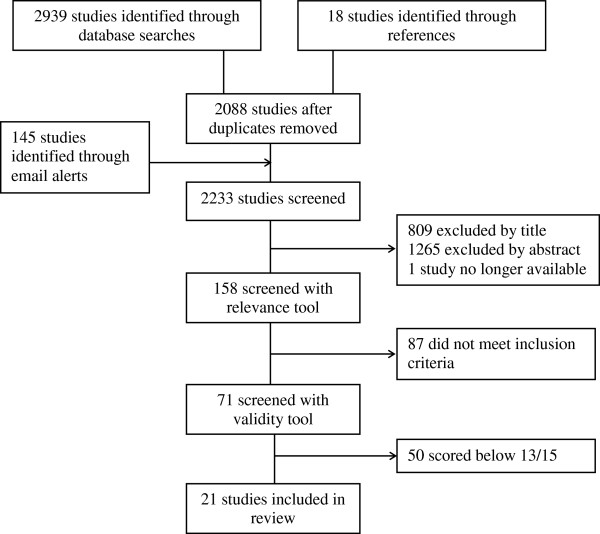
Summary of selection process.

Of the 2233 studies, 809 were excluded by title alone, with an additional 1265 studies excluded following review of their abstracts. A second reviewer randomly selected 10 articles and independently performed the same screening process, reaching the same decisions on exclusion in all 10 cases. Screening and assessing abstracts of studies for relevance to the review yielded 159 potentially relevant articles. One study was no longer accessible, and therefore 158 were assessed with the relevance tool, yielding 71 relevant studies. Inter-rater reliability (kappa) ranged from 0.739 to 0.861 for the reviewing dyads. We applied the validity tool to these 71 studies, with an inter-rater reliability of 0.979, measured via the intra-class correlation. Studies with a score of 13 or higher out of a possible 15 (*n* = 21) were deemed to be of high quality and included in the data extraction (see Figure 1).

### Studies

All studies retained in this review were randomized controlled trials with sample sizes ranging from 52-1297 participants; attrition was less than 24%, and most incorporated multivariate statistical analysis (e.g., analysis of co-variance, multiple regression analysis, or complier average causal effect). Four studies did not pilot test the measures, use well-described tools and/or blind data collectors. Most studies were conducted in the United States (*n* = 15). The relevant outcomes measured were: (a) child abuse and neglect (*n* = 6); (b) developmental delays (*n* = 11); and (c) health assessment (*n* = 10). For each outcome, we report whether the HV intervention had a demonstrable impact. Unless stated otherwise, all control group participants received the usual services offered in their community. Multiple relevant articles arose from the same projects, such as the Healthy Start Program (HSP) and Healthy Families Alaska/New York (see Table 2 for trial characteristics); for sake of clarity these articles are considered individually.

**Table 2:** Characteristics of paraprofessional home visitation evaluation studies included in review

**Author**	**Country**	**N**	Population under study^*^	**Intervention name**	**Frequency of visits**	**Intervention duration**
Aracena et al. [13]^§^	Chile	90	Single pregnant adolescents	-	Monthly	Pregnancy to 1 yr
Barth [14]^≠^	United States	191	At risk women	Child Parent Enrichment Project	Weekly	6 mo (including pregnancy)
Black et al. [15]^§^	United States	130	Children with non-organic failure to thrive	-	Weekly	1 yr
Bugental et al. [16]^≠^	United States	96	Mothers at moderate risk	-	Monthly	Pregnancy to 1 yr
Caldera et al. [17]^≠^	United States	325	Families	Healthy Families Alaska	Varied	2 yrs
Cupples et al. [18]^∞^	Ireland	343	First time mothers	-	Bi-weekly to monthly	Pregnancy to 1 yr
Duggan et al. [19,20]^≠^	United States	643	At risk families	Healthy Start Program	Varied	3-5 yrs
Duggan et al. [21]^≠^	United States	325	At risk families	Healthy Families Alaska	Varied	3 yrs
DuMont et al. [22]^≠^	United States	1297	At risk families Children from poor neighbourhoods	Healthy Families New York-	Weekly to bi-weekly	5 yrs
Grantham-McGregor et al. [23]^§^	Jamaica	129			Weekly	2 yrs
Hamadani et al. [24]^§^	Bangladesh	321	Undernourished children and adequately nourished comparison	Bangladesh Integrated Nutrition Program + psychosocial stimulation	Weekly to bi-weekly	1 yr
Johnson et al. [25]^¥^	Ireland	262	First time mothers with children aged up to 1 yr	Community Mothers’ Programme	Monthly	1 yr
Kartin et al. [26]^§^	United States	78	Substance abusing mothers	Seattle Birth to 3 Program	Weekly to bi-weekly	3 yrs
King et al. [27]^≠^	United States	513	At-risk families	Hawaii Healthy Start Program	Weekly to quarterly	3 yrs
Lee et al. [28]^∞^	United States	502	At-risk adolescent mothers	Healthy Families New York	Bi-weekly	Pregnancy
Le Roux et al. [29]^∞^	South Africa	788	Mother-child-dyads with malnourished child	Philani Child Health and Nutrition Program	Monthly	1 yr
McLaughlin et al. [30]^≠^	United States	428	At-risk pregnant women	-	Not stated	Pregnancy
Nair et al. [31]^≠ ∞^	United States	161	Substance abusing mothers	-	Weekly to bi-weekly	2 yrs
Necoechea [32]^¥^	United States	52	Children at risk for poor school readiness	Home for Parents of Preschool Youngsters	Bi-weekly	15 wks
Scheiwe et al. [33]^§^	United Kingdom	101	Low income mothers	-	Monthly	1 yr

^*^Unless otherwise stated, “at-risk” refers to at-risk for child maltreatment, abuse or neglect.

^§^Study scored 15/15 on validity tool.

^≠^Score of 13-14/15 on validity tool related to attrition >18%.

^∞^Score of 13-14/15 on validity tool related to inadequacies in data collection, e.g., not blinding data assessors.

^¥^Score of 13-14/15 on validity tool related to not using multivariate statistics.

### Child abuse and neglect

Child abuse and neglect was often measured using reports recorded with Child Protective Services (CPS) and/or self-reported behaviours of mothers. All of the studies focused on families deemed at-risk for child abuse. Please see Table 3 for a summary of the outcomes of the studies that assessed child abuse and neglect.

**Table 3:** Results of trials reporting on child abuse and neglect

**Study**	**Results**
Barth [14]	Non-significant
Bugental, et al. [16]	Enhanced group had less harsh parenting and physical abuse compared to other groups
Duggan, et al. [21]	Decreased rate of substantiated child maltreatment within a subset of intervention mothers (non-depressed with moderate to high anxiety)
Duggan, et al. [19]	Non-significant
Duggan, et al. [20]	Non-significant
DuMont, et al. [22]	Reduction in reported minor physical aggression and harsh parenting within a subset of intervention mothers (first time mothers less than 19 yrs of age, enrolled in at less than 30 wks gestation)

Barth [14] evaluated the Child-Parent Enrichment Project for its impact on preventing perinatal child abuse; pregnant women received, on average, 11 home visits over a 6 month period. In general, self-reported measures did not reveal significant differences in the prevention of child abuse between the intervention and control groups. Self-reported measures and lack of blinding of the assessors were seen as methodological weaknesses of this study.

Bugental and colleagues [16] assessed the effectiveness of two types of HV interventions compared to a control group. One intervention group received a program based on the Healthy Start model (called the unenhanced group) while the second group received HV with a cognitive change component (the enhanced group). Child abuse was measured on the basis of harsh parenting style using the self-report Conflict Tactics Scale. Bugental and colleagues [16] found that the enhanced intervention group had less frequent harsh parenting compared to the unenhanced or control groups (*p* = 0.05). As well, the enhanced group mothers were significantly less likely to physically abuse (*p* < 0.05) and least likely to spank/slap their children (*p* < 0.05) compared to the unenhanced or control groups. These findings suggest that enhanced programming (i.e., HV with a cognitive change component) can effectively reduce the frequency and occurrence of harsh parenting among at-risk families. On the other hand, Barth [14] questions the efficacy of paraprofessional services in preventing abuse and neglect in high-risk families because participants in the Child-Parent Enrichment Program experienced no improvement in prevention of abuse.

Duggan, Berlin, Cassidy, Burrell, and Tandon [21] undertook an evaluation of Healthy Families Alaska (HFA), assessing reports on child abuse or maltreatment measured by the number of protective service reports filed. Levels of depression/anxiety and maternal attachment were considered moderators of the impact of HV intervention on child welfare. Among non-depressed mothers with moderate to high anxiety, HV was associated with decreased rates of substantiated child maltreatment (*p* < 0.05). Among mothers who were not depressed, but had high discomfort with trust/dependence, HV was actually associated with increased rates of substantiated child maltreatment. Thus, benefits of this HV intervention seemed to be limited to certain subsets of at-risk mothers where a number of complex factors were at play. Studies by Duggan, Fuddy and colleagues [19] and Duggan, McFarlane and colleagues [20] of the HSP found that there is little impact from paraprofessional services in preventing child abuse and neglect in high-risk families. The researchers surmise that it may be that home visitors are inadequately trained to work with such complex high-risk families, as they were unable to identify family risks and did not provide professional referrals. All the above mentioned studies incorporated large sample sizes, blinded assessors, utilized multiple tools, and ensured study power to detect differences, which can lend credence to the findings.

DuMont and colleagues [22] assessed Healthy Families New York (HFNY) for the program’s effect on child abuse and neglect, as measured by review of CPS records and self-report of mothers over a two-year period. The researchers indicated that no program effects were noted for the sample as a whole, but that differences were detected between subgroups. By the second year of the intervention, the prevention sub-group (first-time mothers less than 19 years old admitted to the study at less than 30 weeks gestation) was less likely to report engaging in minor physical aggression (over the previous year; *p* = 0.02) and harsh parenting behaviours (within the previous week; *p* = 0.02) than was the control group. The “psychologically vulnerable subgroup” (women who were less likely to be first-time mothers, were older, and had a higher rate of prior substantiated CPS reports) were less likely to report acts of serious abuse or neglect compared to the control group at year two (*p* < 0.05). The frequency of these acts was also significantly less than among the control group. DuMont and colleagues [22] suggest that intervening with specific groups of pregnant women can prevent child abuse before it has an opportunity to occur; however, unlike HFNY, assignment of intervention prenatally is not always considered in other large Healthy Families America HV programs.

### Developmental delays

A total of 11 studies, one of which was a thesis, measured impacts related to developmental outcomes of children less than six years of age. Specific developmental outcomes included: (a) psychomotor and cognitive development; (b) child behaviour; and (c) language development. Please see Table 4 for the summary of outcomes for the 11 studies that assess developmental outcomes.

**Table 4:** Results of trials reporting on developmental delays

**Outcome**	**Study**	**Results**
Psychomotor & Cognitive Development	Aracena, et al. [13]	Non-significant
	Black, et al. [15]	Non-significant
	Caldera, et al. [17]	Intervention group more likely to score within normal range of the BSID than control group
	Cupples, et al. [18]	Non-significant
	Hamadani, et al. [24]	Intervention effects on mental development index of the BSID, but not motor development
	Grantham-McGregor, et al. [23]	Intervention effects on development quotient and subscales of locomotor, hand eye coordination, hearing and speech, and performance.
	Johnson, et al. [25]	Intervention effect on developmental stimulation, but not motor development games
	Kartin, et al. [26]	Non-significant
	Nair, et al. [31]	Intervention group had higher scores on psychomotor development index of the BSID than control group
Child Behaviour	Caldera, et al. [17]	Intervention group scored better on the internalizing/externalizing scale of the Child Behavior Check List than control group
	Hamadani, et al. [24]	Intervention benefited cooperation, response-to-examiner, emotional tone and vocalizations.
	Kartin, et al. [26]	Non-significant
Language Development	Aracena, et al. [13]	Non-significant
	Black, et al. [15]	Intervention group showed less decline in receptive and expressive language compared to control group
	King, et al. [27]	Non-significant
	Nair, et al. [31]	Non-significant
	Necoechea [32]	Intervention effect noted for expressive language skills, but not receptive or emergent literacy skills

#### Psychomotor and cognitive development

Over half of the studies (*n* = 6) utilized some version of the Bayley Scales of Infant Development (BSID) to assess psychomotor and cognitive development. Black, Dubowitz, Hutcheson, Berenson-Howards, and Starr [15] undertook a study of an HV program that included weekly visits over a one-year period, conducting analysis on groups of children stratified by age (those < 12 months old and those 12-24 months old). After the 12-month study period, all of the children in the study showed a significant decline in cognitive development overall. However, younger children experienced significantly less decline (*p* = 0.02) compared to age-matched control group children. Differences among the older children were not significant, suggesting that parents of infants may be more receptive to the benefits of an HV intervention compared to parents of toddlers, whose children are undergoing more complex developmental stages. It is important to note that this study may be limited in its generalizability due to the predominantly African American, single mother sample.

The HFAK program was evaluated over a two-year period by Caldera and colleagues [17] on developmental, behavioural and child health outcomes. The researchers found that 18 months after recruitment, children in the intervention group were significantly more likely to score within the normal range on the BSID (mental development index) than control children (*p* < 0.05). The researchers cautioned that families with a low risk for child abuse may be the only to benefit from this program.

Grantham-McGregor, Powell, Walker and Himes [23] assessed effects of nutritional supplementation and psychosocial stimulation (conducted by home visitors) over a two year period with stunted 9 – 24 month old children in Jamaica. Only those findings relating to stimulation (alone and in combination) will be discussed here, as supplementation falls outside the scope of this review. Mothers and children assigned to the stimulation group participated in weekly play sessions led by the community health aides; these sessions were designed to promote the children’s development. The measures of development in this study were based on the Griffiths Mental Development Scales, including four subscales: locomotor, hand-eye coordination, hearing and speech, and performance. The researchers found statistically significant improvements in the first 12 months of the study for the stimulated group in regards to developmental quotient and the subscales of locomotor, hand-eye coordination and performance compared to the control group children (all *p* < 0.01).

Further, over the whole two years of the study [23] significant results continued for stimulated children with respect to developmental quotient and all the subscales (*p* < 0.01). Multiple regression analyses of the final developmental quotient scores revealed that the group of children who received both supplementation and stimulation improved significantly more than the stimulated group (*p* < 0.05). The findings suggest that small improvements in mental development can be seen in stunted children who receive a stimulation intervention alone, however, greater benefits are seen when nutritional supplementation is added to the HV intervention. This study does have two limitations: a small sample size, and the use of a developmental tool which was not standardized for use with the local population.

Hamadani, Huda, Khatun, and Grantham-McGregor [24] conducted a study of developmental outcomes of Bangladeshi children. They measured developmental assessment using the BSID (revised version) before and after 12 months of an HV intervention. Benefits of the intervention on motor development were not significant. They found intervention effects on the mental development index of the BSID (*p* < 0.01), and further, the data were analyzed for children deemed undernourished compared to control group children. Children in the intervention group that were undernourished remained similar to the better-nourished children with respect to mental development on the BSID, but lagged behind on psychomotor development. This study, similar to Grantham-McGregor and colleagues [23], highlights the interacting or moderating effects of nutrition and its impact on overall child development.

Johnson, Howell and Molloy [25] assessed psychomotor and cognitive development using games with one-year-old children in Ireland; the intervention group received a home visit once a month. Mothers were asked how often they played either cognitive (e.g., hide and seek) or motor (e.g., playing with a ball) games with their child and this number was recorded with each game played receiving a score. The number of games was totaled with a higher score indicating children were assessed as more developmentally stimulated. Children in the intervention group were significantly more developmentally stimulated with cognitive games compared to the control group (*p* < 0.01); motor development was not significantly different between groups. A note of caution with these findings is the fact that game playing was used as a means to assess developmental outcomes rather than using a standardized tool.

Nair, Schuler, Black, Kettinger and Harrington [31] compared the psychomotor and cognitive development of 18-month olds with a similar population of substance-abusing mothers. Using the BSID, children in the intervention group who received weekly visits for the first six months of life and then bi-weekly visits up to 24 months had significantly higher scores on the psychomotor developmental index at six months of age (*p* = 0.041) and at 18 months (*p* = 0.01) compared to the control group. The home visits were intended to enhance the mother’s communication with her infant. The researchers suggested there is benefit to using early intervention to improve high-risk children’s psychomotor and mental development.

#### Child behaviour

Caldera and colleagues [17] also assessed children for behavioural outcomes, finding that children in the HFAK program scored more favourably on the internalizing scale (*p* < 0.01) and also on the externalizing scale (*p* < 0.01) of the Child Behavior Checklist compared to control group children. The results from this study show that HFAK was able to reduce problem behaviours in young children, to a degree; other factors related to child behaviours (e.g., maternal depression or partner violence) were not influenced by the HFAK program.

Hamadani and colleagues [24] assessed child behaviour during testing using five 9-point scales. The researchers noted treatment effects for response to the examiner (*p* = 0.01), cooperation with test procedures (*p* = 0.005), emotional tone (*p* = 0.03) and vocalizations (*p* = 0.005); no treatment effect was noted for infant’s activity. This suggests that during testing children in the intervention group benefited in that they were more likely to be willing to engage with the examiner and were more vocal compared to the control group children. It is unclear what the usefulness of these five scales implies on aspects of child behaviour outside of the testing situation within the study.

#### Language development

Five studies considered findings with respect to language development. Black and colleagues [15] used the Receptive/Expressive Emergent Language Scale to assess differences in language development between the younger and older groups of children in their study. Both the younger and older children intervention groups experienced significantly less of a decline (*p* = 0.05) in receptive and expressive language compared to their age-matched control groups.

The study by Necoechea [32] assessed language of three- to four-year-old children using the Peabody Picture Vocabulary Test, Expressive One-word Picture Vocabulary Test-revised, and the Developing Skills Checklist. Testing was done prior to initiation of the Home Instruction for Parents of Preschool Youngsters program, and at the end of the 15-week intervention. Positive treatment effects were noted for the expressive language skills of children (*p* < 0.01) in the intervention group, but no treatment effect was detected for receptive language skills or emergent literacy skills for those same children. The author noted that results should be viewed with caution, as there was substantial variation in the implementation of the intervention, such as number of visits and quality.

### Health assessment

Measures assessed included (a) physical growth; (b) number of hospitalizations, illnesses, or injuries; and (c) up-to-date immunizations. Much of the data collected for these outcomes are from medical records. Ten of the included studies assessed health outcomes. Please see Table 5 for a summary of the health outcomes for each study.

**Table 5:** Results of trials reporting on health assessments

**Outcome**	**Study**	**Results**
Physical growth	Aracena, et al. [13]	Non-significant
	Black, et al. [15]	Non-significant
	Hamadani, et al. [24]	Non-significant
	Lee, et al. [28]	Intervention effect on low birth weight
	Le Roux, et al. [29]	Intervention effect on rehabilitating malnutrition
	McLaughlin, et al. [30]	Non-significant
	Scheiwe, et al. [33]	Non-significant
Hospitalizations, illness, or injuries	Aracena, et al. [13]	Non-significant
	Bugental, et al. [16]	Enhanced group had best health outcomes followed by the unenhanced and control group
	Caldera, et al. [17]	Non-significant
	Duggan, et al. [20]	Non-significant
	Johnson, et al. [25]	Non-significant
	Scheiwe, et al [33]	Intervention group less likely to experience health problems.
Up to date immunizations	Johnson, et al. [25]	Intervention group more likely to receive primary immunizations

#### Physical growth

Aracena and colleagues [13] assessed weight among the one year olds in their study and found no statistical difference between the intervention and control groups. The small sample size (n = 45 in each group) may account for part of this finding. Further, there are other factors to consider when assessing height and weight in young children that may not be amenable to a HV intervention (e.g., biological factors).

Black and colleagues [15] assessed both height and weight for the 12-month duration of their study. They found the HV intervention did not have an impact on children’s growth rates compared to the control group. Hamadani and colleagues [24] also found that the HV intervention they studied had no impact on improving weight or height for age, or weight for height. Unlike Black and colleagues, Hamadani and colleagues found all children experienced a deterioration in weight for height irrespective of which group they were in (nourished, undernourished, control or intervention). This may, in part, be indicative of the socioeconomic conditions in Bangladesh and the impacts such conditions have on quantities and sources of food.

The Lee and colleagues [28] study of the HFNY program is one of two studies with significant findings with respect to physical growth; they also included a measurement of low birth weight (i.e., < 2500 g). The earlier in pregnancy the intervention was initiated the lower the odds were of the mother having a low birth weight baby, indicating a dose–response relationship between HV and low birth weight. Compared to control group mothers, HFNY mothers who enrolled earlier than 30 weeks gestation (5.1% versus 9.8%; *p* = 0.022), at 24 weeks (5.1% versus 11.3%; *p* = 0.008), and at 16 weeks (3.6% versus 14.1%; *p* = 0.008) had significantly fewer low birth weight babies. Further analyses supported a dose–response, with greater benefit conveyed to those families enrolling earlier in pregnancy (i.e., thus receiving seven or more visits) (2.7% versus 7.2%; OR = 0.30; *p* = 0.079). In the Lee and colleagues study, African American women had the greatest reduction in numbers of low birth weight babies (*p* = 0.022) suggesting that aspects of the environment that African American mothers may find themselves are amenable to change and can result in healthier pregnancies.

Le Roux and colleagues [29] evaluated an HV program that focused on improving the nutrition of children less than 5 years of age (average age 18 months). Over the one-year-period of the study, 43% of young children in the intervention group showed an acceptable weight-for-age and faster catch up growth compared to 31% in the control group (*p* < 0.01). Appropriate weights at birth and weight gain into toddler years in children are important as this sets the stage for longer-term health benefits [29]. Findings of this study should be viewed with caution as there was potential for children in most need of supplementation to be steered toward the intervention group despite the intention to randomize participants.

Mclaughlin and colleagues’ [30] study was designed to assess if birth weight was improved when women were enrolled in an HV program prenatally that included a multi-disciplinary team with paraprofessional home visitors. When comparing the intervention group mothers to control group mothers, the researchers found no significant effect of the intervention in reducing the incidence of low birth weight babies. This finding is in contrast to Lee and colleagues’ findings with the HFNY program.

#### Number of hospitalizations, illnesses or injuries

Bugental and colleagues [16] investigated child health as an outcome of their enhanced HV program. As was mentioned previously, they assessed the effectiveness of two types of HV interventions (enhanced and unenhanced) compared to a control group. After completing a health interview with parents, a health score (i.e., frequency of illness, injuries, and feeding problems) was created for each child, where subscales were converted to *z-*scores and summed. Assessment completed at post-program revealed that the three groups were statistically different (*p* = 0.02), with the enhanced HV group receiving the highest level of benefit in improving child health outcomes (i.e., having the fewest health problems).

In a study conducted in Ireland assessing HV impact on children’s hospitalization outcomes, Johnson and colleagues [25] found no significant differences between the intervention and control group. They did report however, that children from the intervention group had significantly longer in-hospital stays (14 days) compared to the control group children (7 days; *p* < 0.05); the researchers provide no explanation for such a peculiar finding. It would seem that this HV program failed to address aspects of various conditions that can lead to the hospitalization of children.

In a four-year follow-up study, Scheiwe, Hardy and Watt [33] report findings relevant to this review that are related to improvements in height and weight, general health, and number of dental caries after a seven-month HV intervention to improve feeding practices. Mothers from both the intervention and control groups reported whether their children had experienced any health problems within the last three months; children in the intervention group were less likely to have experienced any health problems compared to the control group children (*p* = 0.01). All other health-related outcomes were statistically not significant between groups. The researchers caution that the significant findings are hard to explain and are likely only chance findings.

#### Up-to-date immunizations

One study assessed children’s immunization rates. Johnson and colleagues [25] found that significantly more one-year-old children in the intervention group received three of the primary immunizations (these were not listed in the study) compared to the control group (*p* < 0.01). The results suggest that by empowering parents through an HV program, their children benefited both developmentally and by receiving timely immunizations.

In summary, significant improvements as a result of participating in an HV program are noted for particular parent–child groups. First, some children (e.g., those of psychologically vulnerable women) appear more likely to receive beneficial effects (i.e., protection from abuse and neglect) from an HV intervention, particularly when the intervention is initiated prenatally, than others. Second, HV is associated with developmental improvement and is particularly seen for cognition and problem behaviours, and somewhat less consistently for language skills. Third, in terms of health benefits, improvements are seen in birth weight and appropriate weight gain in early childhood (weight-for-age), fewer health problems, and timely immunizations in children. However, not all evaluated HV programs conclusively show beneficial effects on outcomes in socially high-risk children as evidenced by some studies included in this review.

### Implications for practice and future research

On the basis of participating in an HV program, studies reporting no significant benefits are far more prevalent than studies reporting statistically significant benefits. Given the vulnerability of the population and the challenges socially high-risk families encounter, these results are not particularly surprising. The findings from this review tend to point out how difficult it is to change human behaviour, particularly for families that are part of challenging social conditions. While an HV program works to support individual families it can do little to change the context in which socially high-risk families often live.

This review highlights that HV program effectiveness is greatest when: (a) a higher dose of the intervention over a longer period of time is used; (b) mothers are approached prenatally; (c) paraprofessionals are trained adequately to meet the needs of the families they are serving; and (d) the program’s focus is on a particular issue rather than trying to remedy multiple problems. This review addresses the need to assess in detail what is the most beneficial dose of a home visiting intervention in order to produce intended outcomes. Lee and colleagues [29] demonstrated the association between increased number of visits and reduced odds of having a low birth weight baby. It appears that the earlier an HV program is introduced (ideally prenatally) and the more home visits there are (increased exposure to the intervention), the better the outcomes.

Bugental and colleagues [16] utilized three groups for comparison: two variants of HV programs and a control group. They demonstrated that by focusing the HV program to improve a particular issue, in their case prevention of child abuse, the outcomes improved. This supports the notion that an intervention is of greater benefit when it is targeted to specific needs of families rather than trying to make a large number of improvements. However, some of the included studies noted that the complexity of the family situation was too multifaceted to be addressed by the HV intervention [14,17,19,20]. Thus, we suggest that working with multiple risk families poses the question of *where to begin*? Perhaps a future consideration might be to target families with fewer challenges in order to determine if they would be more likely to experience significant benefits from HV programming. Also, no one intervention can meet the needs of every family; it may be better to consider an HV intervention as one part of a bigger system of supports and services for socially at-risk families.

Many of the included studies did not indicate the duration of the home visit or how closely home visitors followed the program model [e.g., [14-18,21,22,24,25]. This has implications for determining the intensity of the intervention required to generate long-lasting benefits. Perhaps future studies could also assess not only the frequency but the length and quality of visits and how these variables influence intended outcomes of HV programs. More research is required that compares the HV intervention a family receives to the actual program model; in this way it would be possible to discover what does and does not work and for whom.

Given that the majority of studies of home visiting effectiveness have failed to demonstrate benefits, it is important to also consider why that might be the case. Two possible explanations are mentioned here. The first relates to training. Many of the HV programs focused on families with multiple risks (i.e., low income, low education, and substance abuse). These stressful family situations may be overwhelming for a paraprofessional to deal with effectively. It is important to note that all the included studies discussed the paraprofessionals’ training and that they were chosen based on the similarity of life circumstances to the families they were serving. Yet, while similarity of life circumstances may facilitate rapport and trust, it may not supplant the need for home visitors to have specific training to help families in crisis.

Another possible explanation involves duration of the program. For some families in difficult circumstances, their stories involve cyclical crises. Changing such stories may require not only the right resources at the right time, but having access to these over a long period of time; perhaps considerably longer than that which is planned for in standard programs. Both of these possibilities relate to the degree of accommodation of the program to the needs of the family. In order to examine these in research, it might be useful to conduct subgroup analyses, stratifying by level of training of home visitor, complexity of needs of family, and length of time in the program. Analysis of HV program effects for families with non-complex needs may provide greater understanding of the capacity of paraprofessional home visiting to effect change in families. If families with complex needs do not appear to benefit from these programs, then efforts to improve their effectiveness or new programs can be initiated and studied to ensure that these families are well served.

Overall, most studies utilized reliable measurement tools (e.g., some version of the BSID). This type of consistency aids in comparing outcomes of various studies. However, none of the included studies examined the impact of the quality of the relationship between the paraprofessional and the family. The potential benefit of this relationship is either not currently being measured or is not amenable to quantification. It is likely that a mixed-methods approach that includes qualitative data, such as interviews, focus groups with program personnel and families, or observations would provide a deeper understanding of *how* HV programs provide benefits for families.

All of the included studies used randomized controlled trials, generally believed to be the gold standard in study design. This is ideal in order to address issues of potential bias and to determine if the intervention truly had an impact or not. However, many of the studies did not clearly articulate how randomization was achieved, which may raise concern regarding selection bias. Further, almost half of the studies had one year or less of follow-up and evaluation. It may be beneficial to consider the long-term effects of HV programs later in childhood; only one study in the review was a follow-up study conducted four years after the HV intervention [33].

Retention of participants is also an issue that requires careful consideration. Almost half of the included studies (*n* = 10 [e.g., [14,16,17,19-22,27,30,31]) had attrition rates of more than 18% of the total sample. Of the studies where attrition was less an issue other factors may have influenced whether a family stayed in the HV program. For example, Hamadani and colleagues [24] included nutritional supplementation in addition to psychosocial stimulation for one group of children; other studies had weekly or bi-weekly visits as part of the intervention [e.g., [15,18,23,24,26,28,32]. Perhaps the frequency of visits and the addition of other incentives improve the likelihood of families remaining in an HV program. What challenges a family faces and why they leave a program are important to consider in order to strengthen HV programs to meet the needs of socially high-risk families.

The aim of this systematic review was to assess the state of the literature regarding the effectiveness of paraprofessional HV programs on child outcomes. The effects of HV programs on family members (e.g., mothers, fathers or siblings) of young children would be an interesting avenue of exploration for future reviews. Other focused systematic reviews could include examination of under-developed countries alone (including consideration of non-English studies), less complex family situations (e.g., those with low income only) or follow-up studies that go beyond six years of age of the study children.

### Limitations

Overall, this review is limited by the articles retrieved. Other research in this area may have been completed, but was not accessed using standard and systematic literature search and retrieval methods utilized here. None of the researchers contacted provided any other work in progress. The findings of this review must be considered in light of the potential for publication bias, selective reporting within studies and methodological limitations found in the included studies; as well as, in the conduct of the review itself. However, the authors took considerable care to ensure the integrity of the review and to be unbiased in their assessment of the included studies through the use of standardized tools.

## Conclusions

This systematic review begins to address a current gap in the research literature by evaluating the effectiveness of paraprofessional HV programs. While this systematic review has shown that HV programs that utilize paraprofessionals often do not have significant effects on disadvantaged families, it does show that young children in these programs show modest improvements in some circumstances. The included studies found that HV intervention programs were associated with decreases in harsh parenting, improved cognition and language development in young children, reductions in low birth weight, improved weight-for-age in young children, and reduction in child health problems. However, findings that were not statistically significant were much more common than significant ones. As discussed, addressing the dose of interventions, approaching women prenatally, focusing programs on improving specific outcomes, making sure paraprofessionals receive adequate training and support, and improving the retention of families all may improve the impacts of HV programs.

## Abbreviations

HV: Home visiting; BSID: Bayley scales of infant development; HFAK: Healthy families Alaska; HFNY: Healthy families New York; HSP: Healthy start program.

## Competing interests

The authors have no financial relationships relevant to this article to disclose. The authors have no conflict of interest to disclose.

## Authors’ contributions

SP was involved in reviewing the articles and extracting the data for the study. SP drafted and was responsible for writing the manuscript. SK managed article retrieval, reviewed all articles and was involved in data extraction. SK also assisted in the write up and formatting of the manuscript. EW performed the literature searches. She was also involved in the initial review of all articles to determine the relevance of the articles and provided assistance with editing the manuscript. DN was involved in reviewing the articles and provided assistance with editing the manuscript. NM contributed to the conception and design of the study, provided assistance with drafting the manuscript and is the Principal Investigator on a project on which the study is based. All authors read and approved the final manuscript.

## Pre-publication history

The pre-publication history for this paper can be accessed here:

http://www.biomedcentral.com/1471-2458/13/17/prepub
